# A surgical case of mediastinal hematoma caused by a minor traffic injury

**DOI:** 10.1186/s13019-020-1065-x

**Published:** 2020-01-10

**Authors:** Katsuyuki Suzuki, Satoshi Shiono, Kazuki Hayasaka, Makoto Endoh

**Affiliations:** Department of Thoracic Surgery, Yamagata Prefectural Central Hospital, 1800, Ooazaaoyagi, Yamagata, 990-2292 Japan

**Keywords:** Mediastinal hematoma, Thyroid bleeding, Minor traffic injury

## Abstract

**Background:**

Mediastinal hematoma rarely occurs after a minor traffic injury.

**Case presentation:**

A woman in her forties was transferred to the emergency room by ambulance due to a traffic accident. Computed tomography (CT) revealed no abnormal findings, and she went home. Two days after the accident, the contrast-enhanced CT was repeated, which revealed cervical and mediastinal hematomas. Because it was possible that there was active bleeding from the right inferior thyroid artery, embolization of the right inferior thyroid artery was performed; however, her condition further deteriorated, so we performed emergency surgery to achieve hemostasis and remove the hematoma. Because of oozing from the right thyroid lobe, we performed right hemithyroidectomy and drainage of mediastinal space and right thoracic cavity. Since there was no bleeding site in the mediastinum, we thought that the mediastinal hematoma was due to bleeding from the thyroid gland. Her postoperative course was uneventful, and she is doing well at 9 months of follow-up after surgery.

**Conclusions:**

It is possible that mediastinal hematoma might be caused by a minor traffic injury**.**

## Background

Most massive mediastinal hematomas are associated with great vessel disruption or major injury [1]. There are a few reports of mediastinal hematoma not associated with great vessel disruption or major injury. Additionally, there have been no reports of massive mediastinal hemorrhage after a minor traffic injury. Because massive mediastinal hematoma due to minor injury is very rare, the indication for surgical intervention is controversial [1–5]. We herein report a very rare surgical case of mediastinal hematoma caused by a minor traffic injury.

## Case presentation

**Fig. 1:**
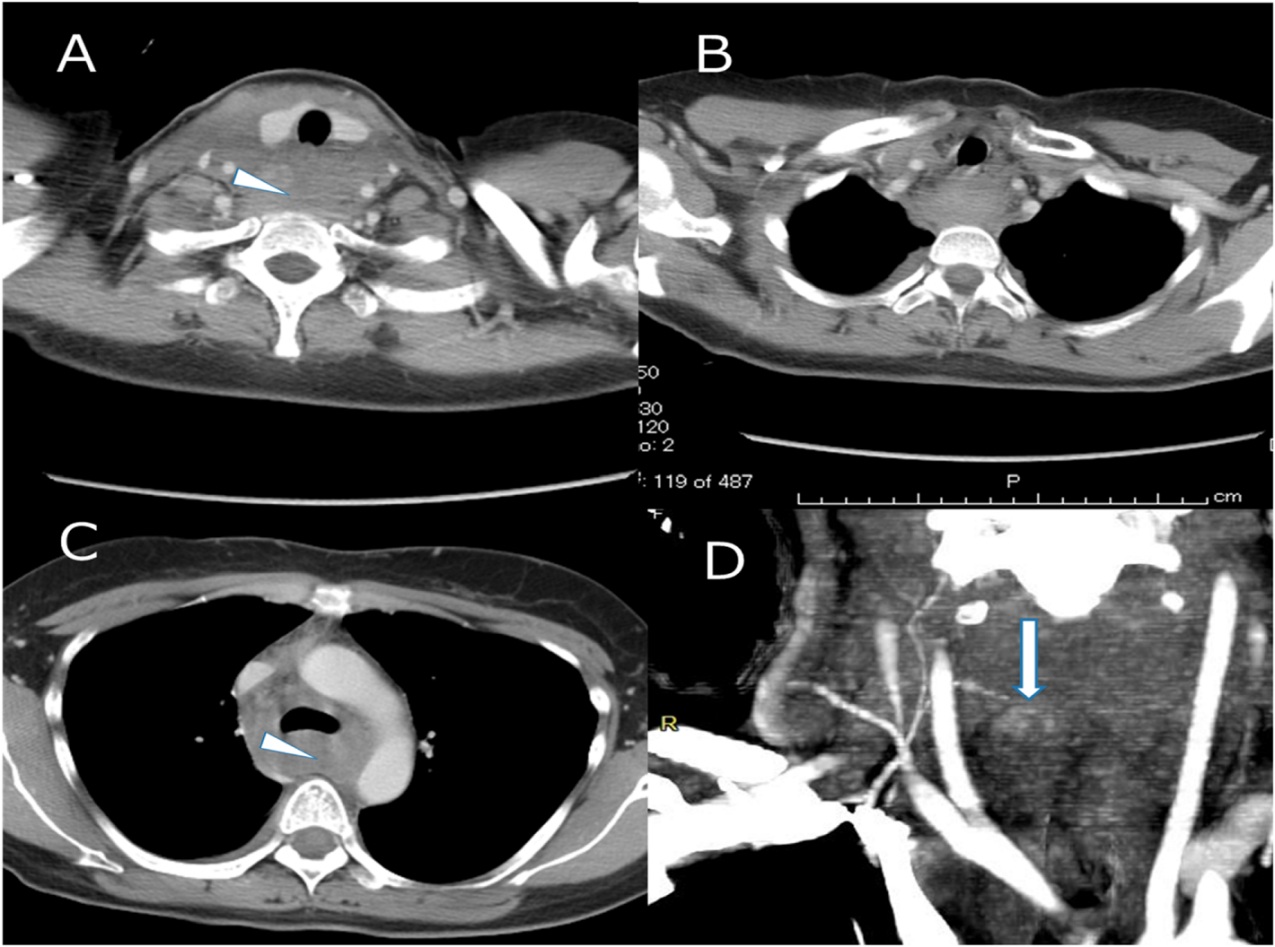
CT findings. **a** A cervical hematoma in the retropharyngeal space. **b** Mediastinal hematoma. **c** A mediastinal hematoma compressing the trachea, esophagus, and superior vena cava. **d** Extravasation of contrast medium (arrow)

**Fig. 2:**
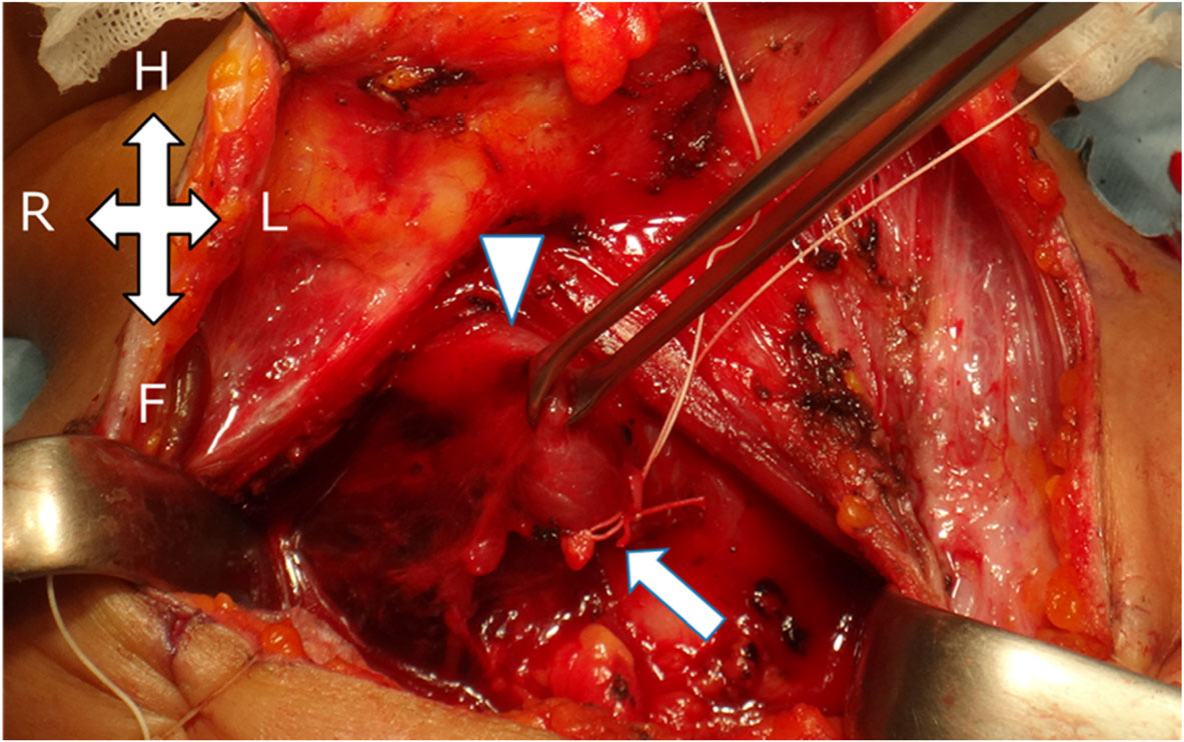
Cervical surgical view. Ligation of the right inferior thyroid artery and vein (arrow) Right lobe of the thyroid gland (arrowhead). In Fig. 2, H: Head, F: Foot, R: Right, L: Left

**Fig. 3:**
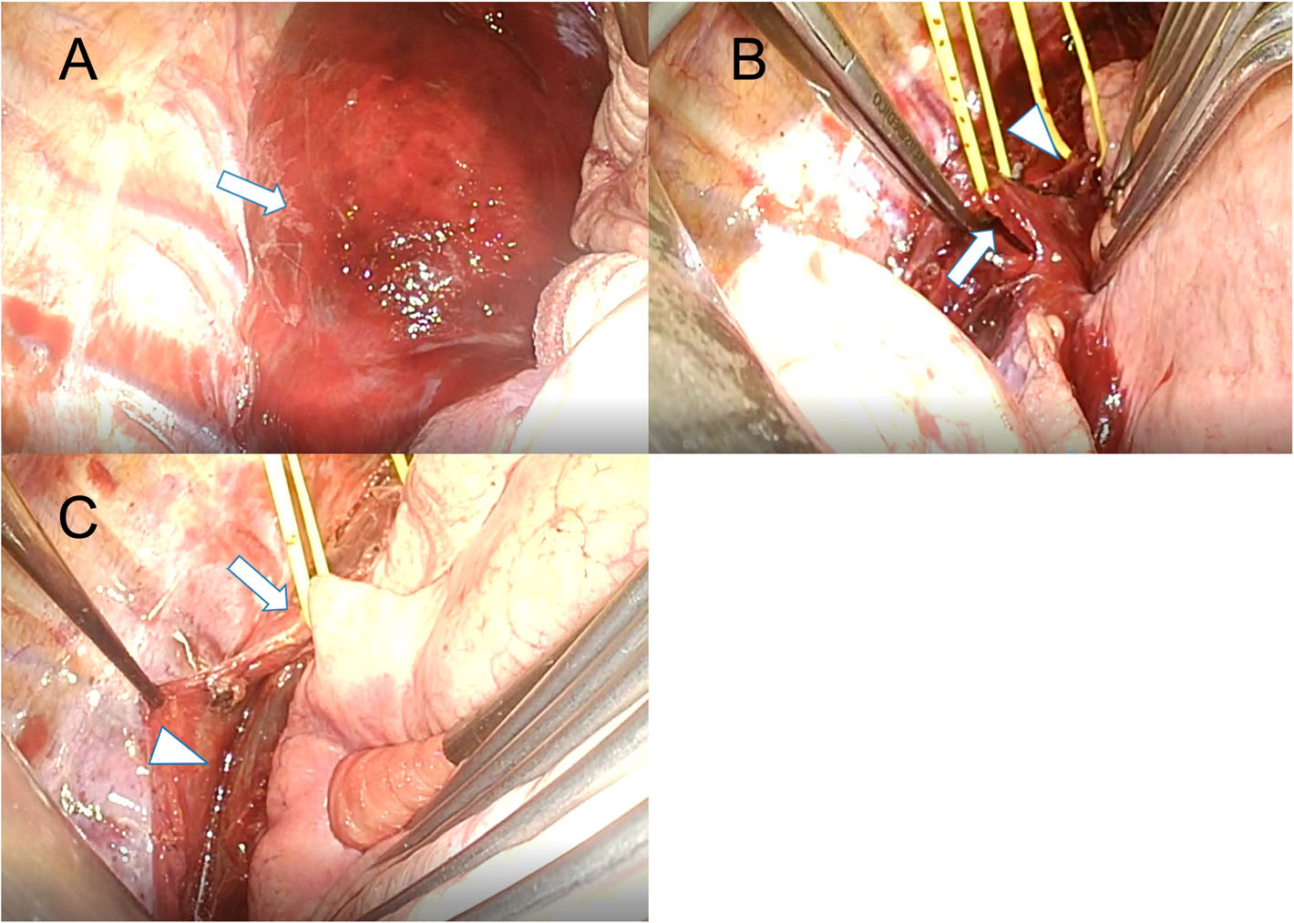
Thoracic surgical view. **a** The hematoma extended from the cervical pleura to the surface of the diaphragm, and there was marked swelling of the mediastinum. **b** The mediastinal pleura was fully opened, and the azygos (arrow) and vagus nerves (arrowhead) were secured. **c** The hematoma between the pre-vertebral lesion and dorsal aspect of the esophagus was removed. Vagus nerves (arrow), Esophagus (arrowhead)

A woman in her forties was transferred to the emergency room by an ambulance due to a traffic accident. While sitting in the driver’s seat of an automobile traveling at 40 km per hour, a lightweight truck traveling at 30 km per hour crashed into the right side of her vehicle. Because of the location of the impact, the front air bag did not deploy. Just after the accident, she complained of left posterior neck pain and slight left chest pain. Since computed tomography (CT) revealed no abnormal findings, she went home. Ten hours after the accident, when she bent back, she felt a sudden sore throat and dyspnea, and the pain continued. On the next day, she visited an orthopedic surgery department, and her condition was diagnosed as cervical sprain. Two days after the traffic accident, she visited the emergency room again due to cervical swelling, dysphagia, chest pain, and severe fatigue. Repeated contrast-enhanced CT revealed a massive retropharyngeal and mediastinal hematoma compressing the trachea, esophagus, and superior vena cava. The CT findings suggested extravasation from the right inferior thyroid artery (Fig. 1). To confirm and embolize the site of bleeding, angiography was performed. However, angiography could not identify the active bleeding point, and we performed the embolization of the right inferior thyroid artery according to the CT findings. Despite embolization, her condition further deteriorated. We decided to perform surgery to obtain hemostasis and drain the mediastinal hematoma. First, we performed surgery of the neck and ligated the right inferior thyroid artery and vein. To stop the oozing from the thyroid gland, a right hemithyroidectomy was performed (Fig. 2). Subsequently, mediastinal drainage and hemostasis was performed through a posterolateral thoracotomy. There was a hematoma mainly at the prevertebral lesion, and mediastinal drainage was carried out (Fig. 3). The mediastinal pleura was fully opened, and the azygos and vagus nerves were secured. The hematoma between the pre-vertebral lesion and the dorsal aspect of the esophagus was removed. Since a site of bleeding in the mediastinum could not be identified, we believed the mediastinal hematoma was due to bleeding from the thyroid gland. Total blood loss was 255 g, and the operative time was 106 min. Because of laryngeal edema, she was extubated on postoperative day 2. After leaving the intensive care unit, her clinical course was uneventful, and she was discharged on postoperative day 14. She is doing well at 9 months of follow-up after surgery.

## Discussion

Mediastinal hematoma without major thoracic injury is reported to be extremely rare [1]. According to previous reports, various causes have been reported: thyroid gland rupture by blunt neck trauma [2, 3], rupture of the inferior thyroid artery by repetitive Valsalva maneuver [1], violent coughing [4], spontaneous rupture of an inferior thyroid artery aneurysm [5], and hemorrhage in a parathyroid adenoma [6]. The thyroid gland has a rich blood supply, and we could not find bleeding from the inferior thyroid artery and mediastinum. Thus, we speculated this patient’s bleeding could have been caused by neck bleeding from the thyroid gland due to increased venous pressure resulting from the traffic injury. Moreover, it is thought that the blood passed through the retropharyngeal prevertebral space, which is the same root as a deep neck infection [2].

Lemke et al. reviewed 34 cases of thyroid gland hemorrhage after blunt neck trauma and reported the delayed onset of symptoms due to bleeding from the thyroid gland, even 24 h after neck trauma [3]. In this case, the patient showed remarkable symptoms 2 days after the traffic accident. At first, we did not suspect that the mediastinal hematoma was due to the minor accident. We subsequently realized that the minor traffic injury may have caused massive mediastinal bleeding. Even after a minor traffic injury, patients who complain of a sore throat or chest pain could have mediastinal bleeding. Such patients should be observed to assure that their condition is stable.

The treatment of mediastinal hematoma depends on the cardiorespiratory conditions [1, 2]. Angiography and embolization are less invasive, and embolization is regarded as an option for treatment. On the other hand, if a patient has severe cardiorespiratory problems due to active bleeding, massive hematoma and tracheal compression, surgical intervention and drainage of the mediastinal hematoma may be necessary. If a patient’s general condition is stable, close monitoring and conservative treatment may be appropriate. In this case, although we adopted a wait-and-see strategy after embolization of the right inferior thyroid artery, her general condition was deteriorating, and CT revealed compression of mediastinal organs including the trachea due to the hematoma; therefore, we decided to perform emergent surgery. As a result, she had a good clinical course. The treatment of mediastinal hematoma should be based on the patient’s status.

## Conclusions

We described a rare case of mediastinal hematoma following a minor traffic injury. Even though such a case is very rare, it should be kept in mind that a minor traffic injury could cause a mediastinal hematoma due to thyroid bleeding.

## Data Availability

Not applicable.

## Abbreviation

CT: Computed tomography
